# Sensor-based digital health technologies to capture endpoints in recent clinical trials: a scoping review

**DOI:** 10.1038/s41746-026-02512-w

**Published:** 2026-04-03

**Authors:** Julia Garcia, Margaret M. Mordin, Bidur Banjara, Cassondra Saande, Katelyn R. Keyloun, Jessica L. Abel, Ari Gnanasakthy, Bill Byrom

**Affiliations:** 1 Health Economics and Outcomes Research Strategy, AbbVie, Irvine, CA USA; 2 RTI Health Solutions, Durham, NC USA; 3 Digital and Data Strategy, AbbVie, Irvine, CA USA; 4 Patient Centered Outcomes Research, AbbVie, Florham Park, NJ USA; 5 eCOA Science, Signant Health, Nottingham, UK

**Keywords:** Biomarkers, Outcomes research, Clinical trials

## Abstract

Sensor-based digital health technologies (DHTs) enable continuous collection of physiological data, sensor-based functional outcomes, and performance outcomes in clinical and real-world settings. However, cross-therapeutic reviews examining sensor-based DHTs as outcome measurement tools rather than interventions in recent pharmaceutical and device trials are lacking, limiting understanding of practical implementation and utility in COA development for use in clinical trials. To address this gap, a scoping review was conducted that encompassed clinical studies that used sensor-based DHTs and were published in MEDLINE, MEDLINE In-Process, and PsycINFO databases from January 2021-December 2023. In total, 48 studies were included, and most (*n* = 38; 79%) collected sensor-based physiological data, with continuous glucose monitoring (CGM) being the most frequent. Additionally, 12 studies (25%) described sensor-based outcomes, such as physical activity and sleep; 2 studies collected both sensor-based physiological data and clinical outcomes. Our findings highlight the use of sensor-based DHTs in clinical research to measure patient outcomes and describe challenges in implementing these technologies in clinical trials.

## Introduction

Advancements in technology have spurred the development, availability, and implementation of digital health technologies (DHTs) in clinical research. These technologies use computing platforms, connectivity, software, and/or sensors to provide efficient data collection and analyses^1^. In this review, we specifically assess the implementation of sensor-based DHTs—such as wearable accelerometers and interstitial glucose monitors—that detect and measure physical or chemical information and use processing systems to translate these data into an output signal that can be remotely transmitted to healthcare providers^2–6^. To support the increasing role of DHTs in clinical research, the US Food and Drug Administration (FDA) and the European Medicine Agency (EMA) established guidelines to promote fit-for-purpose use of DHTs when evaluating medical products^4–6^. The emergence of these sensor-based technologies in clinical trials has afforded several advantages^7,8^, including reducing the burden of data collection on participants, which may improve study enrollment, adherence, and retention^7^. Additionally, sensor-based DHTs collect data at high frequencies, which enables the acquisition of more frequent data for richer insights and may increase the ability to detect change over time. Further, sensor-based DHTs may offer opportunities to measure aspects of health that could not be measured reliably before, such as the measurement of novel endpoints in real-world settings that may be difficult to collect in a clinical setting^7,8^. Lastly, evaluating data collected during patients’ daily lives allows a more patient-centered approach that better reflects an individual’s lived experience^8^. Overall, sensor-based DHTs have evolved as powerful tools that may provide deeper insights into treatment-related changes through high-frequency collection of patient-generated health data in real-world environments^9^.

Sensor-based DHTs can be leveraged in clinical trials to evaluate treatment outcomes, including physiological characteristics that indicate biological processes, or clinical outcomes that measure how a patient functions^9^. For instance, sensor-based DHTs have enabled frequent or continuous measurement of physiological characteristics such as interstitial glucose, which can support hemoglobin A_1c_ (HbA_1c_) endpoints in clinical trials assessing glycemic control in patients with diabetes^10^, and heart rate or blood pressure that can serve as biomarkers within specific contexts of use over the course of a clinical trial^11,12^. Additionally, sensor-based DHTs can be used to actively or passively collect data on performance outcomes or sensor-based functional outcomes (SBFOs), respectively^9,13–15^. Performance outcomes are based on standardized tasks completed by patients according to specific instructions, such as a timed walking test or range of motion exercise, whereas SBFOs are based on passive, non–task-based functional outcomes (e.g., physical movement and sleep) in clinical and real-world settings^9,14,16^. For example, wrist-worn sensor-based DHTs have been used to passively measure physical activity and sleep patterns to evaluate heart conditions, sleep disorders, respiratory disease, obesity, and arthritic conditions^17–23^.

The use of sensor-based DHTs across physiological characteristics, performance outcomes, and SBFOs allows for the collection of a variety of endpoints to generate both indirect and direct evidence of clinical benefit to patients. As an example of a sensor-based biomarker providing indirect evidence of clinical benefit, the Atrial Fibrillation History Feature software, an FDA-approved medical device development tool, pairs with the Apple Watch’s photoplethysmography sensor to detect irregular heart rhythms consistent with atrial fibrillation^24^. This technology can be used to estimate atrial fibrillation burden as a secondary endpoint to evaluate the safety and efficacy of cardiac ablation devices^24^. An example of the potential of sensor-based DHTs to generate more direct evidence of clinical benefit is the use of actigraphy devices to derive time spent in moderate to vigorous physical activity, a recently implemented SBFO measure to support a primary endpoint in a phase 3 clinical study evaluating treatment of patients with pulmonary fibrosis^25,26^.

Given the emergence of new technologies and the evolving nature of the field, sensor-based measures provide many innovative opportunities to assess a variety of concepts in clinical trials. In particular, data collected using performance outcome and SBFO measures can be leveraged as part of an integrated measurement strategy alongside other clinical outcome assessments to inform treatment decision-making and provide a more holistic understanding of the patient experience^9^. Although previous literature has addressed sensor-based DHTs, critical gaps remain. Prior reviews have either (1) focused on DHTs as therapeutic interventions rather than measurement tools^27–31^, (2) examined sensor use within single therapeutic areas (e.g., heart failure^32^, cystic fibrosis^33^, ataxia^34^) rather than across conditions, or (3) emphasized validation and regulatory pathways for acceptance of DHTs in clinical trials^35–37^. No recent review has systematically characterized how sensor-based DHTs are currently deployed as outcome measures across diverse therapeutic areas in pharmaceutical and device clinical trials. This cross-therapeutic perspective is essential because it reveals common implementation patterns that can inform best practices, identifies which sensor technologies have achieved broad adoption, and demonstrates the maturity of the field by showing whether sensor use is isolated to pioneering areas or represents mainstream adoption.

As sensor technology rapidly evolves, a comprehensive review that describes how sensor-based DHTs are currently being used across therapeutic areas to evaluate endpoints and outcomes in pharmaceutical and medical device clinical trials is lacking. Greater understanding of the current use of sensors in clinical research studies—including the concepts assessed via sensor-based DHTs, the types of sensor-based DHTs used to assess these concepts, and the sensor-based outcomes currently being used as primary and secondary endpoints in clinical trials—may help increase the adoption of sensor-based DHTs^9^. Accordingly, this scoping review was conducted with a primary objective of understanding the recent use of sensor-based DHTs to collect patient-generated health data in recent clinical trials of pharmaceuticals and medical devices. In addition to providing an overview of the current use of sensor-based physiological data in clinical trials, we review emerging trends in the use of sensor-based DHTs to generate evidence on clinical outcomes related to patients’ experiences with their disease, condition, or treatment, such as physical functioning or sleep outcomes.

## Results

**Fig. 1:**
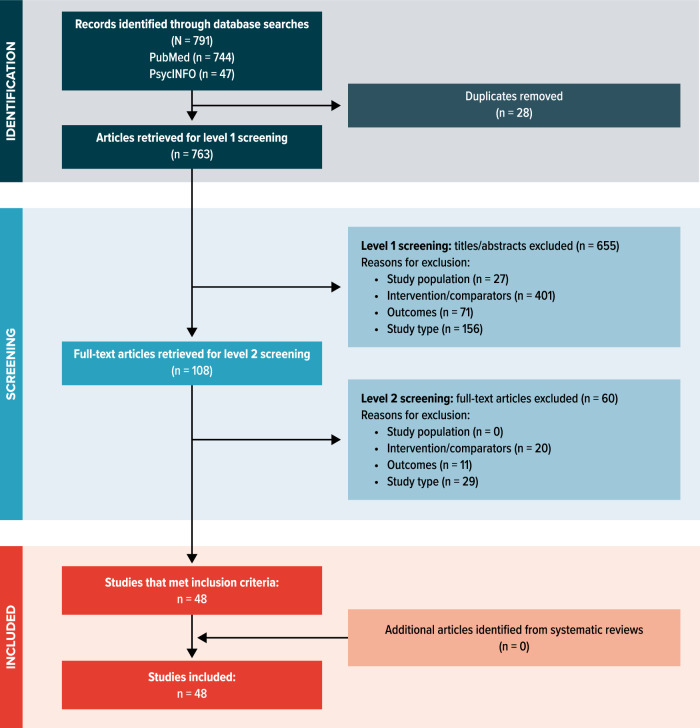
PRISMA flowchart. Among 791 studies identified, 48 studies were included in this scoping review. PRISMA Preferred Reporting Items for Systematic Reviews and Meta-Analyses.

A total of 791 publications were identified through database searches, of which 763 studies were retrieved for screening after the removal of duplicates. Of these, 655 publications were excluded after screening of titles and abstracts, and 60 publications were excluded after the full-text review, resulting in 48 studies that met the inclusion criteria for our review (Fig. 1). Included articles were published from January 2021 to December 2023 and comprised data from 36 countries across 5 continents (Asia, Australia, Europe, and North and South America) and various therapeutic areas, such as endocrinology, cardiology, rheumatology, and neurology. All included studies obtained ethics approval.

### Sensor-based physiological data

**Fig. 2:**
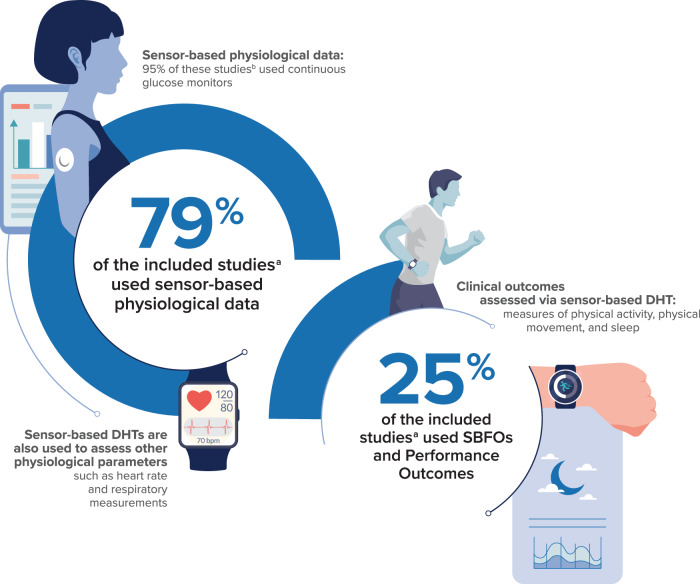
Outcomes measured via sensor-based DHTs in clinical trials. Most of the included studies used sensor-based physiological data captured by continuous glucose monitors, whereas a smaller number of studies used SBFO and Performance Outcome data captured by other sensor-based DHTs. ^a^ *N* = 48; two studies included both sensor-based physiological data and sensor-based clinical outcomes. ^b^ Of the studies that used sensor-based physiological data (*n* = 38), 36 used continuous glucose monitors. DHT digital health technology; SBFO sensor-based functional outcome.

The majority of publications (*n* = 38; 79%) described the use of sensors to measure physiological data, and most (*n* = 36) used continuous glucose monitoring (CGM) in clinical trials conducted primarily in patients with type 1 diabetes (Fig. 2 and Table 1)^38–75^. Of the studies using CGM, 44% (*n* = 16) were conducted among adults only^43–46,49,57,58,62,64–67,69,71,74,75^, 33% (*n* = 12) were conducted among children^40,41,52,55,56,60,61,63,68,70,72,73^, and 22% (*n* = 8) included both adults and children^42,47,48,50,51,54,59,73^. Commonly used CGM systems included the Dexcom G5 or G6 (*n* = 19; 53%)^43,45–47,53,54,56–61,63,65,66,69,72,74,75^ and the Medtronic MiniMed System with the Guardian sensor (*n* = 17; 47%)^40–42,44,48–52,55,62,64,67,68,70,71,73^. All diabetes-related clinical trials identified in this review reported at least 1 of the recommended CGM-derived metrics to be used in combination with HbA_1c_ levels, including time in range (70–180 mg/dL), time below range (<70 mg/dL or <54 mg/dL), time above range (>180 mg/dL or >250 mg/dL), and mean sensor glucose^76^.

**Table 1:** Studies including sensor-based physiological data

Article	Study design/phase	Intervention and comparator	Patient population (medical condition, sample size, age ^a^)	Technology type and name	Sensor wear location	Concept measured and key endpoints	Endpoint position
**CGM studies**							
Beardsall et al.^41^	International, open-label parallel-group, randomized controlled	CGM vs standard clinical practice and measured blood glucose levels	Preterm infants with diabetes (type not specified) *N* = 180 Within 24 h of birth	Internal sensor, Enlite sensor with Guardian 2 Link transmitter to MiniMed 640 G	Lateral thigh	Glucose monitoring ■ Percentage of time glucose level is within a specific range ■ Mean glucose level ■ Sensor glucose variability	Primary and secondary
Biester et al.^42^	Double-blind, randomized, placebo-controlled, crossover	Dapagliflozin vs placebo	Type 1 diabetes *N* = 30 Median (IQR): 17 (12-20) years	Internal sensor, DreaMed Glucositter; Enlite II sensor with MiniLink REAL-Time transmitter	Not reported	Glucose monitoring ■ Sensor glucose time in specific range ■ Average and standard deviation of glucose readings ■ Percentage of glucose readings in specific range	Primary and secondary
Bisio et al.^43^	Nonrandomized, single-arm	CGM with SAP therapy vs CGM with AID	Type 1 diabetes *N* = 15 68.7 (3.3) years	Internal sensor, DexcomG6; wearable, Actiwatch	CGM: abdomen or upper buttocks; Actiwatch: wrist	Glucose monitoring and sleep ■ Time in specific glucose range ■ Mean glucose level ■ Percentage of glucose readings within certain range ■ Total sleep time	Not reported
Bode et al.^44^	Double-blind, 2-period, crossover, randomized controlled	Ultrarapid lispro vs continuous subcutaneous insulin infusion	Type 1 diabetes *N* = 42 47.8 (13.8) years	Internal sensor, MiniMed670G system, Guardian Sensor 3	Back of upper arm or abdomen	Glucose monitoring ■ Mean glucose level ■ Percentage of glucose readings within specific range	Primary and secondary
Boughton et al.^45^	Open-label, multinational, randomized, crossover	HCL vs SAP	Type 1 diabetes *N* = 37 Median (IQR): 68 (63-70) years	Internal sensor, Dexcom G6	Abdomen	Glucose monitoring ■ Proportion of time in specific glucose range ■ Mean glucose level ■ Time in hyperglycemia and hypoglycemia	Primary and secondary
Boughton et al.^46^	Double-blind, multinational, randomized, crossover	Fast-acting insulin aspart vs standard insulin aspart	Type 1 diabetes *N* = 25 38 (9) years	Internal sensor; DexcomG6	Abdomen	Glucose monitoring ■ Proportion of time in specific glucose range ■ Mean glucose level ■ Glucose variability	Primary and secondary
Burnside et al.^47^	Open-label, randomized, controlled	AID vs SAP	Type 1 diabetes *N* = 97 (48 children, 49 adults) Median (IQR): AID, 14.0 (11.0–15.0) years; SAP, 11.0 (9.0-14.5) years	Internal sensor, DexcomG6	Upper buttocks, or abdomen	Glucose monitoring ■ Percentage of time in specific glucose range ■ Mean glucose level ■ Glucose standard deviation	Primary and secondary
Carlson et al.^48^	Open-label single-arm	Advanced HCL (AHCL) system with automated basal (Auto Basal) and automated bolus correction (Auto Correction)	Type 1 diabetes *N* = 157 (39 adolescents and 118 adults) Overall, 38.3 (17.6) years; adolescents, 16.2 (2.1) years; adults 45.6 (14.0) years	Internal sensor, MiniMed AHCL system, Guardian Sensor 3	Abdomen or back of upper arm	Glucose monitoring ■ Time with glucose levels in specific range	Not reported
Chakrabarti et al.^49^	Randomized, crossover	CL vs SAP therapy	Type 1 diabetes *N* = 30 Median (IQR): 68 (64-71) years	Internal sensor, MiniMed 670G systems, Guardian Sensor 3, Medtronic; Wearable, Actiwatch 2	CGM: abdomen or back of upper arm; Actiwatch 2: wrist	Glucose monitoring and sleep ■ Percentage of time in specific glucose range ■ Mean glucose level ■ Total sleep time	Not reported
Collyns et al.^50^	Randomized, open-label, 2-sequence crossover	AHCL system vs SAP therapy	Type 1 diabetes *N* = 59 23.5 years (range, 7-65)	Internal sensor, MiniMed AHCL system, Guardian Sensor 3	Abdomen, back of upper arm, or upper buttocks	Glucose monitoring ■ Percentage of time in specific glucose range ■ Average glucose	Primary and secondary
Cordero et al.^51^	Single-arm, nonrandomized	MiniMed AHCL system	Type 1 diabetes *N* = 176 (109 adolescents, 67 adults) Adolescents, 11.2 (2.5) years; adults, 45.4 (14.8) years	Internal sensor; MiniMed 780 G system; Guardian 4 Sensor	Upper arm	Glucose monitoring ■ Mean glucose level ■ Glucose coefficient of variation ■ Time spent in specific glucose range	Not reported
Daly et al.^74^	Open-label, randomized, crossover	Closed-loop glucose control vs standard multiple daily insulin injection therapy (control)	Type 2 diabetes *N* = 26 59 (11) years	Internal sensor, Dexcom G6	Abdomen	Glucose monitoring ■ Time in specific glucose range	Primary and secondary
Dovc et al.^52^	Double-blind, multinational, 2-period randomized crossover	Faster-acting insulin aspart with hybrid AID vs standard insulin aspart with hybrid AID	Type 1 diabetes *N* = 30 15.0 (1.7) years	Internal sensor, MiniMed 780 G system, Guardian Sensor 3	Upper arm, abdomen, or upper buttocks	Glucose monitoring ■ Percentage of time in specific glucose range ■ Mean glucose level ■ Glucose coefficient of variation	Primary and secondary
Ekhlaspour et al.^53^	Randomized controlled trial	CLC vs SAP therapy	Type 1 diabetes *N* = 168 Range, 14-71 years	Internal sensor, DexcomG6	Upper buttocks or abdomen	Glucose monitoring ■ Percentage of time in specific glucose range ■ Mean glucose level	Not reported
Ekhlaspour et al.^54^	Randomized controlled trial	CLC	Type 1 diabetes *N* = 168 (CLC: *n* = 112; control: *n* = 56) Range, 14-71 years	Internal sensor, DexcomG6	Upper buttocks or abdomen	Glucose monitoring ■ Percentage of time in specific glucose range	Primary
Forlenza et al.^55^	Nonrandomized, open-label	CGM	Type 1 diabetes *N* = 46 4.6 (1.4) years	Internal sensor, MiniMed 670 G system, Guardian 3 Sensor	Not reported	Glucose monitoring ■ Percentage of time in specific glucose range ■ Mean glucose level ■ Glucose coefficient of variation	Secondary
Fuchs et al.^56^	Open-label, multinational, randomized, 2-period crossover	CL vs SAP therapy	Type 1 diabetes *N* = 72 Range, 1–7 years	Internal sensor, DexcomG6	Upper buttocks or abdomen	Glucose monitoring ■ Between-group difference in time spent in target glucose range ■ Mean glucose level	Primary and secondary
Haidar et al.^57^	Open-label, multinational, randomized, 2-period crossover; phase 2	AID vs SAP therapy	Type 1 diabetes *N* = 36 39 (16) years	Internal sensor, Dexcom G5	Abdomen	Glucose monitoring ■ Percentage of time in specific glucose ranges	Primary and secondary
Haidar et al.^58^	Randomized, placebo-controlled, crossover; phase 3	CL therapy ■ Empagliflozin vs placebo SAP therapy ■ Empagliflozin vs placebo	Type 1 diabetes *N* = 27 38 (15) years	Internal sensor, Dexcom G5 or G6	Abdomen	Glucose monitoring ■ Percentage of time in specific glucose ranges between groups	Primary and secondary
Herzig et al.^75^	Open-label, randomized controlled	CL vs standard therapy	Type 2 or other non-type 1 diabetes *N* = 44 CL, 67.3 (15.0) years; standard, 69.6 (9.6) years	Internal sensor, Dexcom G6	Abdomen or upper arm	Glucose monitoring ■ Time in specific glucose range	Primary and secondary
Isganaitis et al.^59^	Randomized controlled trial	CL system vs SAP	Type 1 diabetes *N* = 63 17 (3) years	Internal sensor, Dexcom G6	Abdomen or upper buttocks	Glucose monitoring ■ Time in specific glucose ranges ■ Hypoglycemia and hyperglycemia events	Primary and secondary
Kanapka et al.^60^	Randomized clinical trial	CLC vs SAP	Type 1 diabetes *N* = 100 (CLC: *n* = 78; SAP: *n* = 22) Range, 6–14 years	Internal sensor, Dexcom G6	Abdomen or upper buttocks	Glucose monitoring ■ Time in specific glucose ranges	Primary and secondary
Kariyawasam et al.^61^	Open-label, randomized, controlled, crossover	Diabeloop CL system vs SAP therapy	Type 1 diabetes *N* = 21 Range, 6–12 years	Internal sensor, Dexcom G6	Abdomen or upper buttocks	Glucose monitoring ■ Mean proportion of time spent in hypoglycemia	Primary, secondary, exploratory, and safety
Lee et al.^62^	Randomized, open-label crossover	Fast-acting insulin aspart (faster aspart) vs insulin aspart (IAsp)	Type 1 diabetes *N* = 25 Median (IQR): 48 (37–57) years	Internal sensor, MiniMed AHCL system, Guardian 3	Abdomen or back of upper arm	Glucose monitoring ■ Total percentage of CGM time in specific range	Primary and secondary
Lindkvist et al.^63^	Randomized, single-blind, 2-period, crossover; phase 4	Dual-hormone CL (insulin and glucagon) vs single-hormone CL (insulin)	Type 1 diabetes *N* = 11 14.8 (1.5) years	Internal sensor, Dexcom G6 Wearable, ActiGraph GT9X Link	Abdomen or upper buttocks; wrist	Glucose monitoring ■ Total percentage of time with glucose level in specific range (overnight and during/after exercise)	Primary and secondary
McAuley et al.^64^	Open-label, randomized crossover	CL vs SAP therapy	Type 1 diabetes *N* = 30 67 (5) years	Internal sensor, MiniMed670G system, Guardian 3 sensor	Abdomen or back of upper arm	Glucose monitoring ■ CGM time in specific range	Primary and secondary
Nanayakkara et al.^65^	Open-label, randomized crossover	AAPS automated insulin delivery system vs stand-alone pump therapy	Type 1 diabetes *N* = 20 45.8 (15.9) years	Internal sensor, Dexcom G5, AAPS	Abdomen	Glucose monitoring ■ Difference in the percentage of time in specific glucose range ■ Mean sensor glucose value	Primary and secondary
Nwokolo et al.^66^	Double-blind, randomized, 2-period crossover	HCL with standard insulin lispro vs HCL with ultrarapid insulin lispro	Type 1 diabetes *N* = 28 44.5 (10.7) years	Internal sensor, Dexcom G6	Abdomen	Glucose monitoring ■ Proportion of time in specific glucose range	Primary and secondary
O’Neal et al.^67^	Post hoc analysis of masked CGM data from a randomized, hybrid, closed-loop	HCL-AID system vs standard therapy	Type 1 diabetes *N* = 120 Range, 25–70 years	Internal sensor, Guardian 3	Abdomen or back of upper arm	Glucose monitoring ■ Percentage of time in specific glucose range	Primary and secondary
Pihoker et al.^68^	Prospective, single-arm, nonrandomized	AHCL insulin pump system	Type 1 diabetes *N* = 160 11.3 (2.5) years	Internal sensor, MiniMed 670G system, Guardian 3 sensor	Abdomen, buttocks, or back of upper arm	Glucose monitoring ■ Mean difference of change in HbA_1c_ from baseline ■ Mean change in percentage of time in specific glucose ranges	Primary and secondary
Pinsker et al.^69^	Open-label, randomized, crossover	AID vs SAP	Type 1 diabetes *N* = 35 39 (16) years	Internal sensor, Dexcom G6	Abdomen	Glucose monitoring ■ CGM time in specific range	Primary and secondary
Pulkkinen et al.^70^	Nonrandomized, prospective, single-arm	AHCL system vs retrospective registry controls	Type 1 diabetes *N* = 70 Range, 2–6 years	Internal sensor, MiniMed 780 G system with SmartGuard	Abdomen or upper buttocks	Glucose monitoring ■ Change in time in range	Primary and secondary
Trawley et al.^71^	Post hoc analysis of prospective data from Older Adult Closed-Loop trial	CGM profiles of older adults using SAP therapy compared with consensus CGM targets	Type 1 diabetes *N* = 30 67 (5) years	Internal sensor, MiniMed 670 G system, Guardian 3 sensor	Abdomen or back of upper arm	Glucose monitoring ■ Percentage of time in specific glucose range	Primary and secondary
von dem Berge et al.^40^	Randomized, controlled, 2-step crossover	SAP ■ Without AID ■ With predictive low-glucose management ■ In an HCL system	Type 1 diabetes *N* = 38 8.7 (3.5) years	Internal sensor, MiniMed 670 G system, Guardian 3 sensor	Abdomen or upper buttocks	Glucose monitoring ■ Between-group difference of time spent at specific glucose levels ■ Mean glucose level	Primary and secondary
Ware et al.^72^	Open-label, randomized, crossover	Automated HCL vs SAP	Type 1 diabetes *N* = 74 5.6 (1.6) years	Internal sensor, Dexcom G6	Abdomen or upper buttocks	Glucose monitoring ■ Difference in time in range between treatments	Primary and secondary
Ware et al.^73^	Open-label, multinational, randomized	HCL vs insulin pump (control) therapy	Type 1 diabetes *N* = 133 Range, 6–18 years	Internal sensor, Guardian 3 or Dexcom G6	Abdomen, upper buttocks, or back of upper arm	Glucose monitoring ■ Time in specific glucose range	Secondary
**Non-CGM studies**							
O’Neill et al.^39^	Double-blind, randomized, parallel design; phase 2	2-Hydroxybenzylamine vs placebo	Atrial fibrillation *N* = 162 ≥22 years	Wearable, Apple Watch	Wrist	Atrial fibrillation ■ Atrial fibrillation, atrial flutter, or atrial tachycardia lasting 30 seconds within 28 days following ablation	Primary
Van den Eynde et al.^38^	Randomized, double-blind, placebo-controlled crossover; phase 2 and 3	Elamipretide vs placebo	Barth syndrome *N* = 12 ≥ 12 years	Wearable, AVIVO mobile patient management system	Chest/torso	Functional status ■ Electrocardiography and accelerometry	Secondary

Notes: In most studies, sensor wear location was not reported. For these studies, approved site locations from the sensor manufacturer were reported in the table. The key endpoints listed for each study are not all inclusive and are representative of key concepts measured via sensors. As such, the listed endpoint positions correspond generally to the sensor-based endpoints within each study.

*AAPS* Android Artificial Pancreas System, *AHCL* advanced hybrid closed-loop, *AID* automated insulin delivery, *CGM* continuous glucose monitoring, *CL* closed loop, *CLC* closed-looped control, *HbA*_1c_ hemoglobin A1c, *HCL* hybrid closed-loop, *IQR* interquartile range, *SAP* sensor-augmented insulin pump, *SD* standard deviation.

^a^Age values are mean (SD) unless otherwise noted.

Most of the included studies reporting CGM were conducted to evaluate insulin delivery systems (*n* = 29; 81%)^40,41,43,45,47–51,53–61,64,65,67–75^, such as comparing an open-source automated insulin delivery system with a sensor-augmented insulin pump in patients with type 1 diabetes^47^. Alternatively, 7 studies (19%) assessed the effect of a drug used in combination with CGM within an insulin delivery system^42,44,46,52,58,62,63^, such as evaluating faster-acting insulin versus standard insulin using a hybrid closed-loop delivery system in patients with type 1 diabetes^52^. Regardless of the intervention being assessed, CGMs were used to measure primary endpoints defined by their longitudinal glycemic profiles.

In addition to the studies employing CGM, 2 studies used sensor-based DHT to gather heart rate and respiratory rate data^38,39^. A study protocol by O’Neill et al.^39^ reported the use of the Apple Watch to continually detect and record heart rate and atrial arrhythmias in an atrial fibrillation clinical trial. Primary outcomes included atrial fibrillation, atrial flutter, or atrial tachycardia lasting at least 30 s^39^. In a study by Van den Eynde et al.^38^, sensor-based DHTs collected both physiological data (i.e., heart rate, respiratory rate) and clinical outcomes data (i.e., physical activity and posture) in participants with Barth syndrome. These participants used a wearable AVIVO mobile patient management system for 7 consecutive days after 5 study visits to continuously measure heart rate, respiratory rate, physical activity, and posture, which the authors evaluated for correlation with functional status (secondary endpoints).

### Sensor-based clinical outcomes

Twelve of the included publications (25%) described sensor-based clinical outcomes, in which sensor-based DHTs were used to measure outcomes, such as physical activity, physical movement, and sleep (Fig. 2 and Table 2)^19–21,38,49,77–83^. Of note, 2 of these publications also used sensor-based DHTs to collect physiological data^38,49^. Although sensor-based DHTs were used to assess clinical outcomes across a range of therapeutic areas, most were used in rheumatology (*n* = 4; 33%)^19,38,81^ and cardiology (*n* = 3; 25%)^20,21,77,83^ clinical trials. In addition, all except 2 studies (*n* = 10; 83%) were conducted in adults only^19–21,49,77–79,81–83^. Most sensor-based DHTs were worn on the wrist (*n* = 10; 83%)^19–21,38,49,77,80–83^; other wear locations included the head, upper back, and lower back^78,79^. Finally, half of the studies (*n* = 6) describing sensor-based clinical outcomes collected these sensor-based measures in combination with complementary clinical outcome assessment measures (Table 3)^20,21,38,49,77,83^.

**Table 2:** Studies including sensor-based clinical outcomes

Article	Study design/phase	Intervention and comparator	Patient population (medical condition, sample size, age ^a^)	Technology type and name	Sensor wear location	Concept measured and key endpoints	Endpoint position
Chakrabarti et al.^49^	Randomized, crossover	CL vs SAP therapy	Type 1 diabetes *N* = 30 Median age (IQR): 68 (64–71) years	Internal sensor, MiniMed 670 G systems, Guardian Sensor 3, Medtronic; Wearable, Actiwatch 2	CGM: abdomen or back of upper arm; Actiwatch 2: wrist	Glucose monitoring and sleep ■ Percentage of time in specific glucose range ■ Mean glucose level ■ Total sleep time	Not reported
Conway et al.^78^	Double-blind, randomized, crossover	Low-frequency deep brain stimulation of the subthalamic nucleus vs high frequency	Parkinson’s disease *N* = 14 69.6 (7.5) years	Wearable, Triaxial accelerometer	Headband over occipital protuberance of skull; upper back	Gait rhythmicity ^b^ ■ Rhythmicity of trunk during gait trials ■ Cadence (step/minute); step time (seconds); step time variability (milliseconds)	Secondary
Huhn et al.^82^	Between-subjects, double-blind, randomized, placebo-controlled; phase 2	Placebo vs low-dose suvorexant vs high-dose suvorexant	Opioid use *N* = 38 41.1 (11.2) years	Wearable, ActiGraph GT9X Link	Wrist	Sleep time ■ Total sleep time during a buprenorphine taper and post-taper following buprenorphine discontinuation	Primary
Khandwalla et al.^19^	Randomized, double-blind; phase 4	Sacubitril/ valsartan vs enalapril	Heart failure with reduced ejection fraction *N* = 140 63 (NR) years	Wearable, Philips Actiwatch Spectrum	Wrist	Physical activity and sleep ■ Change from baseline (week 1) to end of double-blind phase (week 8) in mean activity counts during the most active 30 min/day and during sleep	Primary and secondary
Lawrie et al.^83^	Single-blinded, randomized, controlled	Tourniquet during surgery vs no tourniquet during surgery	Total knee arthroplasty *N* = 107 Tourniquet, 62.6 (7.0) years; no tourniquet, 64.0 (6.7) years	Wearable, Fitbit Inspire HR	Wrist	Sleep and physical activity ■ Sleep duration, quality, and disturbances ■ Step count ■ Maximum resting heart rate	Not reported
Leach et al.^20^	Randomized, double-blind, placebo-controlled, pragmatic factorial, crossover; phase 3	Corticosteroids vs placebo	Knee osteoarthritis *N* = 220 Range, 40–85 years	Wearable, Fitbit	Wrist	Physical activity ■ Change in steps from baseline	Primary, secondary, and exploratory
Mitsutake et al.^79^	Prospective, single-blinded, randomized controlled	Transcranial direct current stimulation plus gait training with functional electrical stimulation vs either individual intervention	Subacute stroke *N* = 34 72.5 (11.2) years	Wearable, WalkAide Trunk accelerometer	Lower back	Walking speed and trunk acceleration ^b^ ■ 10-meter walking test	Not reported
Piepoli et al.^81^	Randomized, double-blind, actively controlled, prospective; phase 3	Sacubitril/ valsartan vs enalapril	Heart failure with reduced ejection fraction *N* = 619 66.9 (10.7) years	Wearable, MotionWatch8	Wrist	Physical activity ■ Change from baseline in a mean daily non-sedentary daytime activity after 12 weeks	Primary
Siebert et al.^21^	International, prospective, observational cohort	Guselkumab and interleukin-17 inhibitors	Psoriatic arthritis *N* = 150 ≥18 years	Wearable, ActiGraph CentrePoint Insight Watch	Wrist	Physical activity and sleep patterns ■ Moderate to vigorous physical activity, estimated total calories burned, estimated steps per day ■ Actual sleep time and wake time; number and average length of awakenings during sleep	Not reported
Siren et al.^77^	Randomized controlled, pragmatic single-center, parallel-group, 1:1 superiority	Unicompartmental knee arthroplasty vs high tibial osteotomy	Medial knee osteoarthritis *N* = 100 Range, 45–65 years	Wearable, Withings Move activity watch	Wrist	Physical activity and sleep patterns ■ Number of steps per day ■ Hours of sleep per day	Secondary
Sydenstricker et al.^80^	Cohort, cross-sectional, observational	Stimulant medications (i.e., Adderal, Vyvanse, Concerta, etc.) vs control group	ADHD *N* = 41 Range, 13–19 years	Wearable, GENEActiv accelerometer	Both wrists	Hand movement ■ Fidgeting	Secondary
Van den Eynde et al.^38^	Randomized, double-blind, placebo-controlled crossover; phase 2 and 3	Elamipretide vs placebo	Barth syndrome *N* = 12 ≥12 years	Wearable, AVIVO mobile patient management system	Chest	Functional status ■ Change in accelerometry counts	Secondary

Notes: In studies in which the sensor wear location was not defined, the approved wear locations from the sensor manufacturer were reported in the table. The key endpoints listed for each study are not all inclusive and are representative of key concepts measured via sensors. As such, the listed endpoint positions correspond generally to the sensor-based endpoints within each study.

*ADHD* attention-deficit/hyperactivity disorder, *CL* closed-loop, *IQR* interquartile range, *NR* not reported, *SAP* sensor-augmented insulin pump, *SD* standard deviation.

^a^Age values are mean (SD) unless otherwise noted.

^b^One or more sensors were used to measure performance outcomes.

**Table 3:** Studies combining sensor-based DHT measures with complementary clinical outcome assessment measures

Study	Sensor-based measures	Clinical outcome assessment measures		
Chakrabarti et al.^49^	Sleep outcomes measured via the Actiwatch 2	**■** Pittsburgh Sleep Quality Index ■ Sleep Diary		
Lawrie et al.^83^	Physical activity and sleep outcomes measured via the Fitbit Inspire HR	**■** VAS pain score ■ Oxford Knee score ■ Forgotten Joint score		
Leach et al.^20^	Physical activity outcomes measured by a Fitbit	**■** KOOS ■ PROMIS Pain Intensity		
Siebert et al.^21^	Physical activity and sleep outcomes measured by the ActiGraph CentrePoint Insight Watch	**■** EQ-5D-5L **■** HAQ-DI ■ PsAID-12 ■ PtGA PsA VAS	**■** VAS pain scores ■ BASDAI ■ WPAI:PsA ■ TSQM-9	**■** DLQ ■ PASS
Siren et al.^77^	Physical activity and sleep outcomes measured via the Withings Move activity watch	**■** KOOS ■ VAS pain score ■ Lysholm Knee Scoring Scale ■ Oxford Knee Score		
Van den Eynde et al.^38^	Functional status (e.g., physical activity outcomes, physiological data) measured via the AVIVO mobile patient management system	**■** PROMIS Fatigue score ■ BTHS-SA Total Fatigue score		

*BASDAI* Bath Ankylosing Spondylitis Disease Activity Index, *BTHS-SA* Barth Syndrome Symptom Assessment, *DHT* digital health technology, *DLQI* Dermatology Life Quality Index, *HAQ-DI* Health Assessment Questionnaire-Disability Index, *HR* heart rate, *KOOS* Knee Injury and Osteoarthritis Outcome Score, *PASS* Patient Acceptable Symptom State, *PGA-PsA* Rheumatologist’s Global Assessment of Disease Activity for Psoriatic Arthritis, *PROMIS* Patient-Reported Outcomes Measurement Information System, *PsAID-12* Psoriatic Arthritis Impact of Disease-12, *PtGA PsA* Patient Global Disease Activity for Psoriatic Arthritis, *TSQM-9* 9-item Treatment Satisfaction Questionnaire for Medication, *VAS* visual analog scale, *WPAI:PsA* Work Productivity and Activity Impairment Questionnaire: Psoriatic Arthritis.

Of the 12 publications describing sensor-based clinical outcomes, 10 reported on SBFOs (i.e., non–task-based functional outcomes)^19–21,38,49,77,80–83^, and 2 studies (17%) used sensor-based DHTs to measure performance outcomes^78,79^. Some of the studies describing SBFOs (*n* = 2; 20%) exclusively measured outcomes related to physical activity^20,81^. In a trial conducted in participants with chronic heart failure with reduced ejection fraction, Piepoli et al.^81^ used an accelerometry watch worn continuously during the 14-week study to gather daily non-sedentary daytime physical activity measures to compare sacubitril/valsartan versus enalapril treatment groups. Similarly, Leach et al.^20^ described physical activity measures using a wrist-worn Fitbit to gather step count data for a randomized controlled trial evaluating the benefits of exercise incentives and corticosteroid injections in patients with osteoarthritis of the knee. This study protocol described the continuous collection of step count data over a 32-week study period, with the primary endpoint being change in daily total steps from baseline.

Other studies reporting on SBFOs measured sleep-specific outcomes (*n* = 2; 20%)^49,82^. In a study evaluating the effects of suvorexant on sleep and withdrawal outcomes in individuals undergoing therapy for opioid withdrawal, Huhn et al.^82^ used wireless, wearable electroencephalography and wrist-worn actigraphy to collect sleep outcomes data. Throughout this 8-day study, participants wore an ActiGraph GT9X Link continuously on their nondominant wrist and a 3-lead encephalography DHT each night to collect measures of sleep, with the primary endpoint being total sleep time. In another study, a randomized crossover trial by Chakrabarti et al.^49^ evaluated the effects of closed-loop insulin delivery versus sensor-augmented pump therapy on glycemia and sleep quality in older adults with type 1 diabetes. For this study, participants wore a Philips Actiwatch 2 on their nondominant wrist for 14-day periods at the end of each trial stage to assess sleep measures, with total sleep time and sleep efficiency as endpoints.

Four studies (40%) measured both physical activity and sleep outcomes^19,21,77,83^. Khandwalla et al.^19^ investigated the effect of initiating sacubitril/valsartan on physical activity and sleep in patients with heart failure with reduced ejection fraction using a Philips Actiwatch Spectrum. Accelerometer data were collected continuously during the 18-week study period, and the primary endpoint was change in mean activity counts (accelerations within a time interval) during the most active 30 min per day from baseline to the end of the double-blind phase. A key secondary endpoint was change from baseline in mean activity counts per minute during the patient’s nightly sleep period. The other 3 studies evaluated treatment outcomes of patients with either osteoarthritis^77,83^ or psoriatic arthritis^21^. For example, Siren et al.^77^ described the use of the Withings Move activity watch to compare the number of steps and hours of sleep per day in patients with knee osteoarthritis who received either unicompartmental knee arthroplasty or high tibial osteotomy. In the study of patients with psoriatic arthritis, Siebert et al.^21^ used the ActiGraph CentrePoint Insight Watch to evaluate the effectiveness of guselkumab or an interleukin-17 inhibitor by assessing their physical activity, calories burned, and sleep for up to 3 months while receiving these treatments.

Lastly, one study (10%) used sensor-based DHT to measure fidgeting^80^. In this study, wrist-worn GENEActiv Original accelerometers were used to investigate the effects of stimulant medication on fidgeting in adolescents with attention-deficit/hyperactivity disorder. Participants wore the accelerometers on both the dominant and nondominant wrist during a study visit (3–4 h total wear time), and accelerometer data were extracted during 2 hearing testing sessions (off medication and on medication) to compare the differences in hand movement data between the 2 sessions.

The remaining 2 studies (17%) that included sensor-based clinical outcomes used sensors to measure performance outcomes^78,79^. In a study investigating the effects of high- and low-frequency deep brain stimulation on gait rhythmicity in Parkinson’s disease, Conway et al.^78^ used Noraxon triaxial accelerometers to provide insight into dynamic postural stability while participants performed 4 walking trials along a 14-meter walkway. The accelerometers were affixed to a headband and to participants’ backs and, following the walking trials, raw accelerations were truncated to include 8 continuous gait cycles in the middle of each trial. These data were used to derive measures of trunk rhythmicity, cadence (steps/min), and step time. Similarly, Mitsutake et al.^79^ used a TSND121 wearable trunk accelerometer to evaluate the effects of transcranial and functional electrical stimulation on walking ability in patients with subacute stroke. In that study, the accelerometer was attached to a belt at the spinous process, and trunk accelerometry-based gait characteristics (e.g., walking speed; harmonic ratio [an indicator of the smoothness within a stride]) were measured as participants walked 16 meters at a self-selected speed.

## Discussion

To increase the use of sensor-based DHTs in clinical trials, it is essential to make the field aware of their diffuse implementation. Although other reviews have described the use of DHTs within specific therapeutic areas, this literature review provides a cross-therapeutic assessment of the current landscape of sensor-based DHTs to measure endpoints in clinical trials, providing a valuable resource to the field. Although sensor-based DHTs were used most frequently for purposes of CGM in diabetes clinical trials, sensors were also used across a range of therapeutic areas to gather other physiological data—such as heart rate and respiratory measures—and to assess clinical outcomes focused on physical activity, physical movement, and sleep (Table 4). Most sensors were worn throughout the duration of the clinical trial, which enabled continuous collection of data reflective of the participants’ real-world experiences.

**Table 4:** Summary of sensor-based DHT use across identified studies

Sensor type	Data type	Therapeutic area	Endpoint (physiological/functional)
CGM	**■** Glucose levels in the interstitial tissue	**■** Diabetes	**■** Physiological
Apple Watch	**■** Heart rate	**■** Atrial fibrillation	**■** Physiological
AVIVO mobile patient management system	**■** Heart rate **■** Respiratory rate **■** Physical activity **■** Posture	**■** Barth syndrome	**■** Physiological and functional
Activity watch	**■** Sleep **■** Physical activity **■** Heart rate **■** Step count	**■** Diabetes **■** Heart failure **■** Knee osteoarthritis	**■** Functional
Accelerometer	**■** Trunk rhythmicity **■** Step cadence **■** Walking speed **■** Trunk acceleration **■** Hand movement	**■** Parkinson’s disease **■** Subacute stroke **■** ADHD	**■** Functional
Actigraph	**■** Sleep **■** Physical activity	**■** Opioid use **■** Psoriatic arthritis	**■** Functional

*ADHD* attention-deficit/hyperactivity disorder, *CGM* continuous glucose monitoring, *DHT* digital health technology.

The use of sensor-based DHTs has opened opportunities for data collection from patients in their daily lives outside clinical settings, serving to complement and contextualize other endpoints. For instance, the increasing use of CGM medical devices in clinical studies has complemented HbA_1c_ findings to provide continuous data on the glycemic impact of therapeutic interventions not previously available through HbA_1c_ alone^76^. Additionally, combining data collected via sensor-based DHTs with data collected through clinical outcome assessments enhances our understanding of the functional impact of a disease or treatment^9,19^. Indeed, 6 studies in this review used sensor-based DHT measures of patient functioning together with clinical outcome assessment measures^20,21,38,49,77,83^. For example, in a study protocol by Leach et al.^20^, key outcomes included daily step counts measured via Fitbit activity trackers; the Knee Osteoarthritis Outcome Score to assess the impact of knee osteoarthritis on pain, function, and quality of life; and Patient-Reported Outcomes Measurement Information System (PROMIS) measures to evaluate outcomes such as pain behaviors and pain interference. Similarly, Chakrabarti et al.^49^ assessed sensor-based sleep outcomes in combination with a daily patient-reported sleep diary. Siebert et al.^21^ described exploring associations between electronic patient-reported outcomes with sensor-based activity and sleep data collected using a wearable actigraph. Additionally, Van den Eynde et al.^38^ used machine-learning models to assess whether wearable sensor-based DHT data (e.g., physical activity outcomes) were correlated to patient-reported outcome assessments, such as the PROMIS fatigue score. In these examples, sensor-derived insights into patients’ physical activity and sleep provide additional context to clinical outcome assessments evaluating pain, functioning, and sleep outcomes, facilitating a more holistic approach to understanding patients’ experiences with their disease, including the impact of their symptoms or the effects of treatment.

Sensor-based DHTs have the potential to become an integral part of data collection in clinical trials to enhance our understanding of the effects of new interventions through the lens of patients’ experiences, particularly through the innovative use of sensor-based DHTs to gather data on outcomes related to patient functioning, such as physical activity and sleep. In this review, we observed that a wide range of clinical trial endpoints related to physical activity and sleep were reported (Table 5), which illustrates the ability to derive specific, patient-focused outcomes via sensor-based DHTs while also underscoring potential complexities in categorizing these data. Despite the importance of physical activity and sleep to overall health^84–86^—and the impact of disease on these outcomes^87,88^—we found the number of studies currently using sensor-based measures of physical activity and sleep to be relatively low. This may be related to challenges in the adoption of these technologies and the validation of sensor-based DHT measures in clinical trials. Notably, concern of contradictory data (e.g., inconsistent findings from patient-reported outcome and SBFO measures) has been highlighted in studies assessing perceived versus objective sleep, underscoring the possibility that patient-reported outcomes and sensor-based DHTs may measure different concepts that should be used collectively for thorough assessments of sleep outcomes^89^.

**Table 5:** Summary of measures used to assess sleep and physical activity

Measure	Sensor location	Concept	Endpoint	Sensor-based DHT
Passive data collection (SBFO)	Wrist	Sleep	**■** Total sleep time **■** Sleep quality **■** Sleep disturbances **■** Sleep and wake time **■** Number and average length of awakenings during sleep **■** Hours of sleep per day	**■** Actiwatch 2 **■** ActiGraph GT9X Link **■** Philips Actiwatch Spectrum **■** Fitbit Inspire HR **■** ActiGraph CentrePoint Insight Watch **■** Withings Move activity watch
		Physical activity	**■** Activity counts change from baseline **■** Step count **■** Maximum resting heart rate **■** Change in steps from baseline **■** Mean daily non-sedentary daytime activity **■** Moderate to vigorous physical activity **■** Estimate total calories burned **■** Estimated steps per day	**■** Philips Actiwatch Spectrum **■** Fitbit Inspire HR **■** ActiGraph CentrePoint Insight Watch **■** Withings Move activity watch **■** Fitbit **■** MotionWatch8
	Chest	Physical activity	**■** Change in accelerometry counts	**■** AVIVO mobile patient management system
Active data collection (Performance Outcome)	Lower back	Physical activity	**■** 10-meter walking test	**■** WalkAide Trunk accelerometer
	Skull and upper back	Physical activity	**■** Gait rhythmicity of trunk during gait trials **■** Cadence, step time, and step time variability	**■** Triaxial accelerometer

Notes: The endpoints listed for each study are not all inclusive and are representative of key concepts measured via sensors.

*SBFO* sensor-based functional outcome.

Across all sensor-based DHTs, practical and regulatory concerns remain a challenge for their implementation into clinical trials^7,35,36,90^. These barriers include additional burdens on clinical trial sites (e.g., cost and challenges related to data handling); data acceptance by payers in health technology assessment decision-making; possible variability in passively collected data from different sensor-based DHT models or following software updates, which could lead to heterogeneity in the evidence generated (i.e., data quality issues); and developing and validating sensor-based measures and endpoints/biomarkers^7,35,36,90^. As significant time and cost are associated with using sensor-based DHT in clinical trials, investigator training on these technologies can often be insufficient, potentially hampering efficient data collection and interpretation^36^. A lack of adequate training can also negatively affect participant compliance (i.e., improperly worn and/or managed sensor-based DHT), which can impact data quality^36^. Although there have been successful regulatory endorsements of the use of sensor-based DHT endpoints in clinical trials—including the EMA’s acceptance of stride velocity 95th centile (SV95C) as a valid endpoint measuring peak physical activity performance in patients with Duchenne muscular dystrophy^91^; the FDA’s acceptance of moderate to vigorous physical activity measured with DHT as the primary endpoint in a pivotal fibrotic interstitial lung disease study^25,26,92^; and the EMA’s acceptance of the PROactive composite endpoint, which includes sensor-derived physical activity measures alongside patient-reported outcomes in patients with chronic obstructive pulmonary disease^93,94^—there have been no sensor-based biomarkers or clinical outcome assessments that have received FDA approval as a drug development tool as of July 2024^35^. To gain regulatory approval of sensor-based DHTs in clinical trials, investigators must first identify a clinical concept of interest that is meaningful to patients and/or a new measure that fills an unmet need, followed by demonstrating that the selected sensor-based DHT is fit-for-purpose (e.g., is appropriate for the intended trial population, has the required technical and performance specification to reliably measure the proposed concept, has supporting systems for data collection and processing)^4,35,95^. These steps require a significant evidentiary burden that can be expensive and time consuming, and even if approval is achieved, it is not guaranteed that the accepted biomarker or clinical outcome assessment can be used as a primary or secondary endpoint in a clinical trial, which can deter investigators from seeking regulatory approval^35^.

Although FDA approval of sensor-based DHT endpoints is a challenge, many sensor-based DHTs have received FDA clearance through the 510 (k) Premarket Notification process. For example, the Dexcom G6 CGM, AVIVO mobile patient management system, Actigraph Centrepoint Insight Watch, MotionWatch, and Apple’s electrocardiograph software and atrial fibrillation feature for the Apple Watch, all of which are described in this review, have received FDA clearance^96^. This level of clearance provides a basis for consumer confidence in these devices to maintain and promote health^97^. As this review shows, these commercially available, FDA-cleared sensor-based DHTs are commonly used in clinical trials across therapeutic areas; however, it is important to note that the granting of clearance for personal health tracking does not always correlate with sufficient accuracy and precision for use in clinical trials for regulatory decision-making. Although this review did not aim to specifically identify and evaluate FDA-cleared and/or FDA-approved devices, future work is warranted to explore this aspect of sensor-based DHTs in clinical trials.

In addition to gathering foundational evidence that supports sensor-based measures as reliable and valid, it is essential that the levels of change detected by sensor-based DHTs correspond to meaningful clinical benefit. In this review, while half of the included studies using sensor-based measures of patient functioning gathered complementary clinical outcome assessment data, the studies reporting on sensor-based clinical outcomes did not provide evidence of the meaningfulness of these outcomes from the patient perspective. Although clinical trial endpoints are routinely defined and assessed through measurements of statistical significance, these quantitative measurements may not reflect meaningful change to the patient, and a mixed methods approach using quantitative and qualitative data may better reflect the overall treatment benefit experienced by the patient^13,98,99^. Thus, to facilitate a more holistic understanding of a patient’s experience, future studies should link sensor-based clinical outcomes to concepts meaningful to patients (e.g., via qualitative evidence collected through concept elicitation or cognitive debriefing). Additionally, results should be interpreted with reference to a meaningful change threshold, missing data should be measured, and the handling of missing data should be described^1,2,100^. Indeed, a recent review by Tackney et al.^37^ highlights these and other key areas that should be considered to properly validate and implement digital outcome measures for use in clinical trials. Taken together, these points underscore the need for a framework that provides practical, clinical guidance related to the selection and use of sensor-based DHTs in clinical trials.

A limitation of this study is that, to capture the most recent and relevant information, the inclusion dates cover a 3-year period from 2021 to 2023. Although this restriction may have missed earlier clinical trials using sensor-based DHTs, this is the first literature review we are aware of that comprehensively evaluates the use of sensor-based DHTs in recent clinical trials. Additionally, our focus was the use of sensor-based DHTs to assess the outcomes of pharmaceutical or medical device interventions within clinical trials, whereas studies assessing other types of interventions (e.g., medical nutrition therapy, lifestyle interventions) were excluded. Studies that used sensor-based DHTs as an intervention or studies that were designed to develop, validate, or evaluate adherence to sensor-based DHTs in clinical trials were also excluded from this review. As such, the emerging use of sensor-based DHTs may be more expansive than the studies included in this review. However, these findings highlight the wide variety of endpoints and outcomes currently collected via sensor-based DHTs in clinical trials. Given the increasing popularity of decentralized clinical trials, the use of sensor-based DHTs is likely to increase.

Overall, the versatility of sensor-based measures within clinical research enables continuous capturing of a variety of key outcomes inside and outside the clinic and across a wide range of therapeutic areas. This scoping review highlights the broad utility of sensor-based DHTs in recent clinical trials to assess physiological data and clinical outcomes, the potential challenges in leveraging sensor-based DHTs to gather patient-generated health data as part of clinical research, and the need to ensure that sensor-based clinical outcomes are meaningful to patients. As new technologies emerge and the field evolves, there is a need for alignment on a framework outlining evidentiary requirements for the development and implementation of sensor-based measures in clinical research, applying approaches across the measurement science and digital health disciplines. Such a framework will support data generation in a way that is reliable, informs therapeutic decision-making, and provides maximal value to patients. Although this evidentiary framework could improve validation and implementation of sensor-based DHTs in clinical trials, it is important to note the lack of sensor-based DHT data used for regulatory decision-making. Reducing the gap between evidentiary qualification and regulatory approval could maximize the utility of sensor-based DHT in clinical trials.

## Methods

This scoping review followed the Preferred Reporting Items for Systematic Reviews and Meta-analyses (PRISMA) guidelines^101^ (Supplementary Table 1) to identify studies that describe sensor-based measures used in recent pharmaceutical or medical device clinical trials. The literature search, screening, and data extraction were based on a predefined review protocol. Literature searches were conducted in MEDLINE, MEDLINE In-Process (via the PubMed platform), and PsycINFO to identify studies published in English from January 2021 through December 2023. Searches were first performed in November 2023, and searches were repeated in January 2024 to fully capture 2023 publications. The search strategy included Medical Subject Heading (MeSH) terms for PubMed and free-text terms as described in Supplementary Tables 2 and 3.

All identified studies were reviewed for eligibility using the predefined inclusion and exclusion criteria presented in Table 6. Briefly, eligible studies included pharmaceutical or medical device clinical trials using sensor-based DHT to evaluate specific concepts (e.g., physical activity) or endpoints; studies focusing on sensor-based DHT development, validation, and adherence were excluded. These criteria were applied at 2 phases of article screening, each performed by a single researcher: (1) screening of titles and abstracts for eligibility and (2) screening of full-text articles. Additionally, bibliographies of relevant systematic literature reviews and meta-analyses were reviewed to identify any studies not identified in the database searches.

**Table 6:** Inclusion and exclusion criteria

	Inclusion criteria	Exclusion criteria
Population	**■** Any patients participating in a clinical trial using sensor-based DHT (i.e., sensor-based technology that includes digital signal acquisition and/or remote monitoring capabilities)	**■** No sensor-based DHT included
Intervention	**■** Any pharmaceutical or medical device	**■** Behavioral intervention (without pharmaceutical or device) **■** Sensor-based DHT as intervention
Comparators	**■** Any	**■** Not applicable
Outcomes	**■** Type of sensor (e.g., electrochemical) **■** Concepts assessed via sensor-based measures (e.g., physical activity) **■** Endpoints using sensor-based outcomes	**■** Sensor-based DHT included but not contributing to a specific study endpoint **■** Adherence to sensor-based DHT
Study design	**■** Clinical trials (phase 1–4) **■** Pre-approval medical device studies (e.g., pivotal and feasibility studies) **■** Reviews and systematic reviews/meta-analyses ^a^	**■** Retrospective studies **■** Sensor-based DHT validation studies **■** Sensor-based DHT development studies **■** Editorials, letters, commentaries, expert opinion

Note: The inclusion and exclusion criteria were applied at level 1 and level 2 screening.

*DHT* digital health technology.

^a^Systematic reviews and meta-analyses were not directly included but were used for identification of primary studies not previously identified.

Data were extracted from each source by a single researcher and were verified through quality control measures by a second researcher who was not involved in the primary data extraction. Any uncertainties were resolved by a third researcher who did not perform the primary data extraction. Extracted data were summarized and synthesized on the basis of the type of sensor-based measure(s) reported in the publications: sensor-based physiological data or sensor-based clinical outcomes. Sensor-based physiological data were defined as any type of physiological data (e.g., CGM-derived time spent in specific glucose range, heart rate) collected via a sensor-based DHT worn by clinical trial participants. Sensor-based clinical outcomes were used as an umbrella term for measures collected via sensor-based DHTs that describe or reflect patient functioning (e.g., measures of physical functioning captured through an activity tracking DHT; these included SBFOs and performance outcomes).

## Supplementary information

Supplementary Information

## Data Availability

All data generated or analyzed within this literature review are included in this article and its supplementary information files.
