# Supplementary material for: Sensor-based digital health technologies to capture endpoints in recent clinical trials: a scoping review
Source: NPJ Digit Med. 2026 Apr 3;9:417. doi: 10.1038/s41746-026-02512-w (PMC13230522; doi:10.1038/s41746-026-02512-w)
Supplement: Supplementary file 1 — Supplementary Information [file 41746_2026_2512_MOESM1_ESM.pdf]

SUPPLEMENTARY INFORMATION

Sensor-Based Digital Health Technologies to Capture Endpoints in Recent Clinical Trials: A Scoping Review

Supplementary Table 1. Preferred Reporting Items for Systematic reviews and Meta-Analyses extension for Scoping Reviews (PRISMA-ScR) Checklist

SECTION	ITEM	PRISMA-ScR CHECKLIST ITEM	REPORTED ON PAGE #
**TITLE**			
Title	1	Identify the report as a scoping review.	1
**ABSTRACT**			
Structured summary	2	Provide a structured summary that includes (as applicable): background, objectives, eligibility criteria, sources of evidence, charting methods, results, and conclusions that relate to the review questions and objectives.	2
**INTRODUCTION**			
Rationale	3	Describe the rationale for the review in the context of what is already known. Explain why the review questions/objectives lend themselves to a scoping review approach.	3-5
Objectives	4	Provide an explicit statement of the questions and objectives being addressed with reference to their key elements (e.g., population or participants, concepts, and context) or other relevant key elements used to conceptualize the review questions and/or objectives.	5-6
**METHODS**			
Protocol and registration	5	Indicate whether a review protocol exists; state if and where it can be accessed (e.g., a Web address); and if available, provide registration information, including the registration number.	16
Eligibility criteria	6	Specify characteristics of the sources of evidence used as eligibility criteria (e.g., years considered, language, and publication status), and provide a rationale.	16-17
Information sources*	7	Describe all information sources in the search (e.g., databases with dates of coverage and contact with authors to identify additional sources), as well as the date the most recent search was executed.	16
Search	8	Present the full electronic search strategy for at least 1 database, including any limits used, such that it could be repeated.	Supplementary Information pages 5-8
Selection of sources of evidence†	9	State the process for selecting sources of evidence (i.e., screening and eligibility) included in the scoping review.	16-17
Data charting process‡	10	Describe the methods of charting data from the included sources of evidence (e.g., calibrated forms or forms that have been tested by the team before their use, and whether data charting was done independently or in duplicate) and any processes for obtaining and confirming data from investigators.	17

SECTION	ITEM	PRISMA-ScR CHECKLIST ITEM	REPORTED ON PAGE #
Data items	11	List and define all variables for which data were sought and any assumptions and simplifications made.	17
Critical appraisal of individual sources of evidence§	12	If done, provide a rationale for conducting a critical appraisal of included sources of evidence; describe the methods used and how this information was used in any data synthesis (if appropriate).	n/a
Synthesis of results	13	Describe the methods of handling and summarizing the data that were charted.	17
**RESULTS**			
Selection of sources of evidence	14	Give numbers of sources of evidence screened, assessed for eligibility, and included in the review, with reasons for exclusions at each stage, ideally using a flow diagram.	32 (Figure 1)
Characteristics of sources of evidence	15	For each source of evidence, present characteristics for which data were charted and provide the citations.	Tables 1 and 2
Critical appraisal within sources of evidence	16	If done, present data on critical appraisal of included sources of evidence (see item 12).	n/a
Results of individual sources of evidence	17	For each included source of evidence, present the relevant data that were charted that relate to the review questions and objectives.	Tables 1 and 2
Synthesis of results	18	Summarize and/or present the charting results as they relate to the review questions and objectives.	6-11
**DISCUSSION**			
Summary of evidence	19	Summarize the main results (including an overview of concepts, themes, and types of evidence available), link to the review questions and objectives, and consider the relevance to key groups.	11-13
Limitations	20	Discuss the limitations of the scoping review process.	15
Conclusions	21	Provide a general interpretation of the results with respect to the review questions and objectives, as well as potential implications and/or next steps.	16
**FUNDING**			
Funding	22	Describe sources of funding for the included sources of evidence, as well as sources of funding for the scoping review. Describe the role of the funders of the scoping review.	18

JBI = Joanna Briggs Institute; PRISMA-ScR = Preferred Reporting Items for Systematic reviews and Meta-Analyses extension for Scoping Reviews.
* Where *sources of evidence* (see second footnote) are compiled from, such as bibliographic databases, social media platforms, and Web sites.
† A more inclusive/heterogeneous term used to account for the different types of evidence or data sources (e.g., quantitative and/or qualitative research, expert opinion, and policy documents) that may be eligible in a scoping review as opposed to only studies. This is not to be confused with *information sources* (see first footnote).
‡ The frameworks by Arksey and O'Malley (6) and Levac and colleagues (7) and the JBI guidance (4, 5) refer to the process of data extraction in a scoping review as data charting.
§ The process of systematically examining research evidence to assess its validity, results, and relevance before using it to inform a decision. This term is used for items 12 and 19 instead of "risk of bias" (which is more applicable

to systematic reviews of interventions) to include and acknowledge the various sources of evidence that may be used in a scoping review (e.g., quantitative and/or qualitative research, expert opinion, and policy document).

From: Tricco AC, Lillie E, Zarin W, O'Brien KK, Colquhoun H, Levac D, et al. PRISMA Extension for Scoping Reviews (PRISMAScR): Checklist and Explanation. Ann Intern Med. 2018;169:467–473. doi: 10.7326/M18-0850.

Supplementary Table 2. PubMed Literature Search Strategy

Search No.	Search Terms	No. of Articles
**Biometric Monitoring Devices**		
#1	"Routinely Collected Health Data"[Majr] OR "Accelerometry"[Majr] OR "Smartphone"[Majr] OR "Mobile Applications"[Majr] OR "Actigraphy"[Majr] OR "Fitness Trackers"[Majr] OR "Polysomnography"[Majr] OR “continuous monitoring”[Title/Abstract] OR device*[Title] OR “activity monitor*”[Title/Abstract] OR “activity tracker*”[Title/Abstract] OR “fitness tracker*”[Title/Abstract] OR “closed-loop”[Title/Abstract] OR accelerometry[Title/Abstract] OR accelerometer*[Title/Abstract] OR actigraphy[Title/Abstract] OR actimetric[Title/Abstract] OR actimetry[Title/Abstract] OR somnograph*[Title/Abstract] OR “sleep monitor*”[Title/Abstract] OR polysomnograph*[Title/Abstract] OR “smart phone*”[Title/Abstract] OR smartphone*[Title/Abstract] OR “mobile application*”[Title/Abstract] OR “mobile app*”[Title/Abstract] OR “portable software app”[Title/Abstract] OR “portable software apps”[Title/Abstract] OR “portable software application”[Title/Abstract] OR “portable software applications”[Title/Abstract] OR “portable electronic app”[Title/Abstract] OR “portable electronic apps”[Title/Abstract] OR “portable electronic application”[Title/Abstract] OR “portable electronic applications”[Title/Abstract] OR “digital health technology”[Title/Abstract] OR “ecological momentary assessment”[Title/Abstract]	265,864
**Sensor-based Wearable Devices**		
#2	#1 AND (Wearable Electronic Devices[Majr:NoExp] OR wearable*[Title/Abstract] OR sensor[Title/Abstract] OR sensors[Title/Abstract] OR smartwatch*[Title/Abstract] OR “smart watch*”[Title/Abstract] OR wristworn[Title/Abstract] OR “wrist-worn”[Title/Abstract] OR Applewatch[Title/Abstract] OR “Apple watch”[Title/Abstract] OR Fitbit[Title/Abstract] OR Garmin[Title/Abstract] OR SleepScore[Title/Abstract] OR RT3[Title/Abstract] OR PKG[Title/Abstract] OR GT3x[Title/Abstract] OR E4[Title/Abstract] OR MC10[Title/Abstract] OR Ax3[Title/Abstract] OR Bodymedia[Title/Abstract] OR ActivPal[Title/Abstract] OR CamNTech[Title/Abstract] OR “Smart patch”[Title/Abstract:~3] OR “Smart patches”[Title/Abstract:~3] OR “wearable patch”[Title/Abstract:~3] OR “wearable patches”[Title/Abstract:~3] OR “electronic patch”[Title/Abstract:~3] OR “electronic patches”[Title/Abstract:~3] OR biosensor*[Title/Abstract] OR “bio sensor*”[Title/Abstract] OR “smart ring*”[Title/Abstract] OR “Fitness Tracker*”[Title/Abstract] OR “Smart Bandage”[Title/Abstract:~3] OR “Smart Bandages”[Title/Abstract:~3] OR hearable*[Title/Abstract] OR “Smart Cloth*”[Title/Abstract] OR "Mobilise-D"[Text Word] OR "proactive instrument"[Title/Abstract:~8] OR "proactive instruments"[Title/Abstract:~8])	29,801

Search No.	Search Terms	No. of Articles
**Study Type**		
#3	#2 AND ("Clinical Trials as Topic"[Mesh] OR trial*[Title] OR “clinical stud*”[Title/Abstract] OR “pivotal trial*”[Title/Abstract] OR “pre-approval study”[Title/Abstract] OR “pre-approval studies”[Title/Abstract] OR “feasibility stud*”[Title/Abstract] OR “phase two”[Title/Abstract] OR “phase 2”[Title/Abstract] OR “phase II”[Title/Abstract] OR “phase three”[Title/Abstract] OR “phase 3”[Title/Abstract] OR “phase III”[Title/Abstract] OR "Clinical Trial"[Publication Type])	2,400
**Exclusions**		
#4	"Animals"[Mesh] NOT "Humans"[Mesh]	5,324,094
#5	"Comment"[Publication Type] OR "Letter"[Publication Type] OR "Editorial"[Publication Type] OR "Case Reports"[Publication Type] OR “case report*”[Title] OR “case stud*”[Title] OR “case series”[Title] OR “case histor*”[Title]	4,683,541
**Total**		
#6	#3 NOT (#4 OR #5)	2,365
#7	#6 AND English[Language]	2,353
#8	#7 AND ("2021/01/01"[Date - Publication] : "2023/12/31"[Date - Publication])	**744**

Supplementary Table 3. PsycINFO Literature Search Strategy

Search No.	Search Terms	No. of Articles
**Biometric Monitoring Devices**		
S1	MJ ("Routinely Collected Health Data" OR "Accelerometry" OR "Smartphone" OR "Mobile Applications" OR "Actigraphy" OR "Fitness Trackers" OR "Polysomnography") OR TI (device* OR "continuous monitoring" OR activity N/1 monitor* OR activity N/1 tracker* OR fitness N/1 tracker* OR "closed-loop" OR accelerometry OR accelerometer* OR actigraphy OR actimetric OR actimetry OR somnograph* OR sleep N/1 monitor* OR polysomnograph* OR smart N/1 phone* OR smartphone* OR mobile N/1 application* OR mobile N/1 app* OR "portable software app" OR "portable software apps" OR "portable software application" OR "portable software applications" OR "portable electronic app" OR "portable electronic apps" OR "portable electronic application" OR "portable electronic applications" OR "digital health technology" OR "ecological momentary assessment") OR AB (device* OR "continuous monitoring" OR activity N/1 monitor* OR activity N/1 tracker* OR fitness N/1 tracker* OR "closed-loop" OR accelerometry OR accelerometer* OR actigraphy OR actimetric OR actimetry OR somnograph* OR sleep N/1 monitor* OR polysomnograph* OR smart N/1 phone* OR smartphone* OR mobile N/1 application* OR mobile N/1 app* OR "portable software app" OR "portable software apps" OR "portable software application" OR "portable software applications" OR "portable electronic app" OR "portable electronic apps" OR "portable electronic application" OR "portable electronic applications" OR "digital health technology" OR "ecological momentary assessment")	74,269
**Sensor-based Wearable Devices**		
S2	MJ "Wearable Electronic Devices" OR TI (wearable* OR sensor OR sensors OR smartwatch* OR smart N/1 watch* OR wristworn OR "wrist-worn" OR Applewatch OR "Apple watch" OR Fitbit OR Garmin OR SleepScore OR RT3 OR PKG OR GT3x OR E4 OR MC10 OR Ax3 OR Bodymedia OR ActivPal OR CamNTech OR Smart N/3 patch* OR wearable N/3 patch* OR electronic N/3 patch* OR biosensor* OR "bio sensor*" OR "smart ring*"OR "Fitness Tracker*" OR Smart N/3 Bandage* OR hearable* OR "Smart Cloth*" OR "Mobilise-D" OR proactive N/8 instrument*) OR AB (wearable* OR sensor OR sensors OR smartwatch* OR smart N/1 watch* OR wristworn OR "wrist-worn" OR Applewatch OR "Apple watch" OR Fitbit OR Garmin OR SleepScore OR RT3 OR PKG OR GT3x OR E4 OR MC10 OR Ax3 OR Bodymedia OR ActivPal OR CamNTech OR Smart N/3 patch* OR wearable N/3 patch* OR electronic N/3 patch* OR biosensor* OR "bio sensor*" OR "smart ring*"OR "Fitness Tracker*" OR Smart N/3 Bandage* OR hearable* OR "Smart Cloth*" OR "Mobilise-D" OR proactive N/8 instrument*)	13,544
S3	S1 AND S2	3,957

Search No.	Search Terms	No. of Articles
**Study Type**		
S4	MA "Clinical Trials as Topic" OR TI (trial* OR clinical N/1 stud* OR pivotal N/1 trial* OR “pre-approval study” OR “pre-approval studies” OR feasibility N/1 stud* OR “phase two” OR “phase 2” OR “phase II” OR “phase three” OR “phase 3” OR “phase III”) OR AB (clinical N/1 stud* OR pivotal N/1 trial* OR “pre-approval study” OR “pre-approval studies” OR feasibility N/1 stud* OR “phase two” OR “phase 2” OR “phase II” OR “phase three” OR “phase 3” OR “phase III”) OR PT "Clinical Trial"	71,495
S5	S3 AND S4	133
**Exclusions**		
S6	MA "Animals" NOT "Humans"	198,547
S7	PT ("Comment" OR "Letter" OR "Editorial" OR "Case Reports") OR TI (case N/1 report* OR case N/1 stud* OR “case series” OR case N/1 histor*)	2,267
S8	S6 OR S7	200,814
**Total**		
S9	S5 NOT S8	133
S11	S10 AND **Limiters** - English	133
S12	S11 AND **Limiters** - Publication Date: 20210101-20231231	**47**
